# Reliability and validity of Polar Team Pro measurements in running at different velocities in an indoor setting

**DOI:** 10.3389/fspor.2023.1165801

**Published:** 2023-05-25

**Authors:** Roland van den Tillaar, Fredrik Gaustad Pettersen, Pål Lagestad

**Affiliations:** Department of Sports Sciences, Nord University, Levanger, Norway

**Keywords:** training monitoring, GPS, artificial intelligence, inertial sensors, IMU

## Abstract

The purpose of this study was to test the reliability and validity of Polar Team Pro measurements of velocity, acceleration, and distance covered in a rectangular run at different intensities in an indoor setting. In two test sessions, 10 women (age 15.7 ± 0.4 years, body mass 61.3 ± 5.3 kg, body height 1.69 ± 0.07 m) performed 100 m runs at different intensities, ranging from 8 to 18 km/h. The 100 m runs were performed on a rectangular track at an indoor handball facility. The main finding revealed that Polar Team Pro underestimated the running distance and velocity (10%–15% at 10 km/h), especially at higher speeds (15% and 6% at 15 and 18 km/h, respectively). Between test days, coefficients of variance varied from 4.2% to 12.4%, when measuring at different speeds. However, a significant difference was found for the two runs only at 15 km/h between the two test days. It was concluded that Polar Team Pro underestimated the running distance and velocity when measuring a rectangular run at different speeds in an indoor setting, especially at higher speeds. This underestimation is probably caused by the inaccuracy of the inertial measurement unit algorithm that calculates the distance, as body height influences the distance and velocity measurements. The variability between the different units is, thereby, also influenced, causing variable coefficients of variance between the sensors. Test–retest variability was acceptable. Based on the findings of this study, practitioners should be cautious when measuring speed and distance using Polar Team Pro Sensors in indoor settings, as these measurements are underestimated with increasing speed.

## Introduction

Performing in sports at a high level during the whole year requires regular training and monitoring of the training load to avoid injuries (1). There are different ways to control the training load during each training session. One is monitoring the total distance and distances at different intensities during each training session (2, 3). These measurements are easy to monitor in athletics, where athletes run at prescribed distances at different intensities. However, in team sports like soccer and handball, these measurements are much more difficult to perform during training sessions and games. Therefore, in the last two decades, measurement systems like Catapult and ZXY have been developed to monitor these distances and intensities using the Global Positioning System (GPS) and accelerometers. These systems have been shown to measure distance and intensity accurately (1). However, they are relatively expensive and use a local position measurement system under outdoor and indoor conditions.

Polar Team Pro is a low-cost system that also uses GPS and accelerometers. It also claims to accurately measure distances at different intensities in outdoor and indoor situations. Thus, under outdoor conditions, Polar Team Pro uses GPS, a system that is not functional under indoor conditions. Two studies (4, 5) have been conducted to measure the validity and reliability of Polar Team Pro under outdoor conditions. First, Akyildiz et al. (4) compared the accuracy and reliability of two Polar Team Pro units for interunit reliability with GPSports as the reference standard to determine concurrent accuracy in the total distance and distances at different velocities. They observed acceptable interunit reliability (<5% typical error of the mean) and concluded that Polar Team Pro was suitable for tracking team sport variables. Furthermore, the Polar Team Pro units were accurate under the same conditions. However, Polar Team Pro is not advised to be used interchangeably with GPSports for quantifying distances covered at higher speeds. Second, Huggins et al. (5) assessed the validity and reliability of Polar Team Pro units in measuring the total distance and velocity under outdoor conditions, but during linear running and a team sport simulation circuit among 15 male soccer athletes. They showed that validity and reliability measures in terms of the total distance had <5% error at all velocities. Furthermore, the validity of the device in measuring velocity was significantly different (*p* < 0.05) at all velocities between the 40 m and the 100 m runs, with effect sizes ranging from trivial to small. Despite the trivial to large effect sizes for the validity of the total distance, the Polar Team Pro units demonstrated good reliability during linear and team sport simulation circuit movements during outdoor testing.

The validity of Polar Team Pro was tested during an indoor situation by Fox et al. (6), who investigated the sensor during continuous locomotive and change-of-direction tasks at low, medium, and high intensities. They found a low agreement between the sensor and the measured speed and distance indoors. However, it was not clearly mentioned if they calibrated the system before using it outdoors to link the GPS-measured data with the inertial measurement unit (IMU) sensor for accurate IMU measurements indoors, as the manufacturer specifies. Furthermore, the validity was measured only at three different intensities per participant, which could lead to missing information. Moreover, as the authors (6) stated, no reliability measurements were conducted, which are important to investigate if the reliability between the different units over several days is acceptable in an indoor setting. This information is very important for athletes, trainers, and embedded scientists for monitoring the training load in indoor sports, e.g., handball and futsal, to avoid overtraining for a competition. Therefore, this study aimed to test the reliability and validity of Polar Team Pro measurements of velocity and distance at different intensities in an indoor setting. Based on the findings of Fox et al. (6) on the continuous locomotive and Change of direction (COD) tasks using Polar Team Pro indoors, it was hypothesized that running speeds would be underestimated.

## Methods

### Participants

A total of 10 female handball players (age 15.7 ± 0.4 years, body mass 61.3 ± 5.3 kg, body height 1.69 ± 0.07 m), who are playing on the national junior league level and free from any injuries or health conditions, performed running tests. Informed consent was obtained prior to testing from all subjects and parents, with the approval of the Norwegian Center for Research Data (NSD), and conformed to the latest revision of the Declaration of Helsinki.

A repeated-measures design was used to test the reliability and validity of Polar Team Pro, in which, in two test sessions, actual 100 m runs (reference distance) at different intensities were performed on a rectangular track at an indoor facility. The total distance, distance, and velocity at different intensities were used as variables and compared with control-measured 100 m distances. Reliability was tested according to systematic bias, random error, and retest correlation.

### Procedure

**Figure 1:**
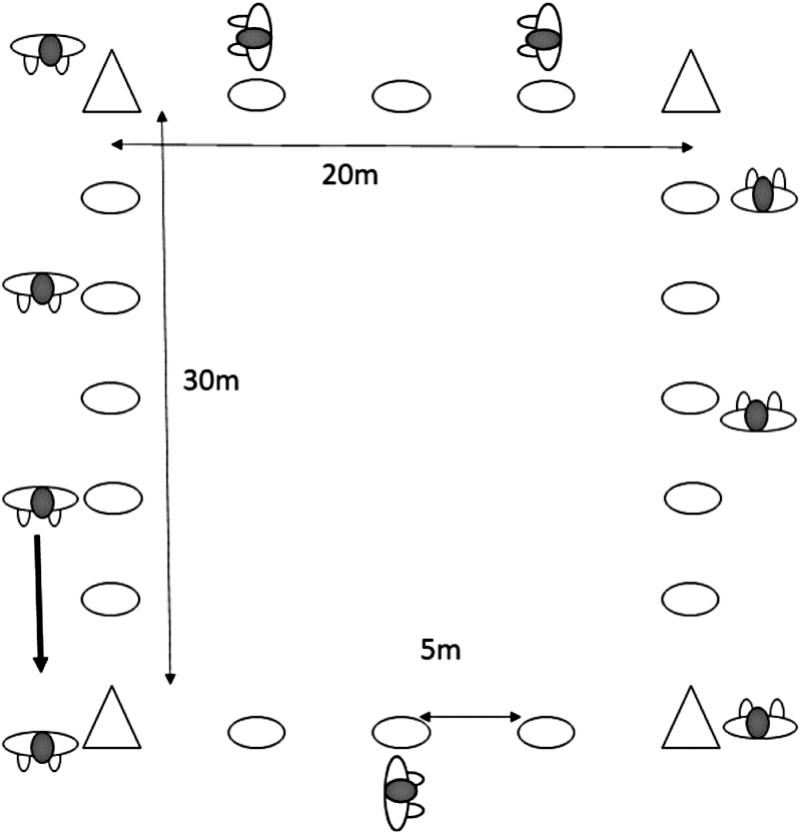
Experimental setup: a rectangle (20 m × 30 m) track marked with cones at an indoor facility.

Before using Polar Team Pro (Polar Electro, Kempele, Finland) indoors, the system was used in two training sessions, i.e., warmup and running outdoors. The manufacturer recommends calibrating the inertial movement units with GPS signals. Each participant wore the same Polar Team Pro strap (Polar Electro, Kempele, Finland) positioned on the center of the chest at the Xiphoid process level at each session to calibrate the sensors. After calibration, the system was used during indoor handball training sessions for 4 months before the testing days. On the test and retest days, the same sensor was used by each participant. After an individualized warmup of 5 min, each of the participants started at a cone after each other, with the same distance between them on a rectangular track (20 m × 30 m) marked with cones on each 5 m. The distances were control-measured with a measuring tape to millimeter accuracy. On a signal, they started to jog at a prescribed tempo. Each participant had to hold the same distance from the other participants the whole time (Figure 1). The starting tempo was 8 km/h. Every 25 m, a signal was given to ensure that the participants had the correct pace. After 200 m, the pace was increased to 10 km/h for 200 m. It was followed with 2 m × 100 m at 12, 15, and 18 km/h with some rest between the runs. See Table 1 for full details on the running time and rest between each run. In the rest periods, the participants had to stand still to prevent the system from detecting extra meters. A total of 1,000 m had to be covered.

**Table 1:** Running protocol with the distances, intensities, and rest between runs.

Running condition (km/h)	8	10	12	15	18
Actual distances (m)	2 × 100	2 × 100	2 × 100	2 × 100	2 × 100
Time per 100 m (s)	45	36	30	24	20
Rest between runs (s)	0	0 (18 s after the second run)	30 (after the second run)	36	40
Total time (min) accumulated	1.5	3	4.5	6.5	8.5

For every occasion for each participant, the total distance and the velocities at different intensities (3–6.9, 7–10.9, 11–14.9, 15–18.9, >19 km/h) were automatically calculated by the accelerometers in the Polar Team Pro system, with sampling frequencies of 10 Hz together with the average velocity, which should be 7.66 m/s for this protocol. Furthermore, each 100 m from the raw data at a different intensity was calculated based on the prescribed time for each 100 m on each occasion and used in further analysis.

### Statistical analysis

Validity and reliability were tested in three different ways, i.e., systematic bias, random error, and test–retest, similar to the validation studies of Balsalobre-Fernandez et al. (7) and van den Tillaar and Ball (8). The systematic bias was calculated for the total distance, average velocity during the whole session per speed category, and each 100 m between prescribed distances, velocities, and actual measured parameters. These biases were calculated by using a paired sampled *t*-test on each testing occasion. A 2 (runs 1 and 2) × 5 (speed) ANOVA with repeated measures was performed between the measured distances per speed and per run to investigate whether the measured distances changed between runs and velocity. A random error was estimated as the standard deviation between the sensors/mean of the sensors × 100 for the different variables using the coefficient of variance (CV), in which a CV under 10% is considered good (9). The test–retest was expressed by an intraclass correlation coefficient (ICC) for the total distances and distances per speed, measured by each sensor during the two testing days. Furthermore, a one-way ANOVA for repeated measures was performed between the first and the second tests for each variable (velocity and distance). Where the sphericity assumption was violated, the Greenhouse–Geisser adjustments of the *p*-values were reported. The effect size was evaluated with eta partial squared, where 0.01 < *η*^2^ < 0.06 constitutes a small effect, 0.06 <*η*^2^ < 0.14 a medium effect, and *η*^2^ > 0.14 a large effect (9). The interpretations of ICC were that values below 0.5 indicated poor reliability, between 0.5 and 0.75 moderate reliability, between 0.75 and 0.9 good reliability, and above 0.9 excellent reliability (10). The level of significance was set at *p* ≤ 0.05, and all data are expressed as mean ± SD. Statistical analysis was performed in SPSS version 27.0 (SPSS, Inc., Chicago, IL, United States).

## Results

The total running distances, average velocity of the whole protocol, and running distances in the different speed categories (directly measured and calculated by Polar Team Pro) were significantly different from the actual distances (*t* ≥ 2.45, *p* ≤ 0.037). A *post-hoc* comparison showed that the total distance was underestimated on each testing day by, on average, 50 and 75 m. Furthermore, the distance covered at 0–10.99 km/h was significantly overestimated by ≈94 m, while the distance covered at 11 km/h was underestimated by 150–170 m. The average velocity was underestimated by 0.66 m/s (Table 2), while the peak velocity reached over 18 km/h.

**Table 2:** Average (±SD) total distance, maximal and average velocity, and distance per different velocity measured with Polar Team Pro.

Test	Total distance (m)	Maximal velocity (m/s)	Average velocity (m/s)	Speed (3–10.9 km/h)	Speed (11–19 km/h)
Test day 1	950 ± 64	19.9 ± 2.5	6.8 ± 0.5	494 ± 71 m	453 ± 130 m
Test day 2	925 ± 86	18.8 ± 2.2	7.0 ± 0.7	495 ± 61 m	426 ± 135 m
Actual distance (m)	1,000			400 m	600 m
CV test day 1 (%)	6.7		6.7	14.4	28.7
CV test day 2 (%)	9.4		9.4	12.3	31.6

CV, coefficient of variance.

Speed categories 3–6.9 and 7–10.9 km/h, and categories 11–14.9, 11–18.9, and >19 km/h were taken together since some distances were not accounted for. All measured values were significantly different from the actual values on a *p* < 0.05 level.

**Figure 2:**
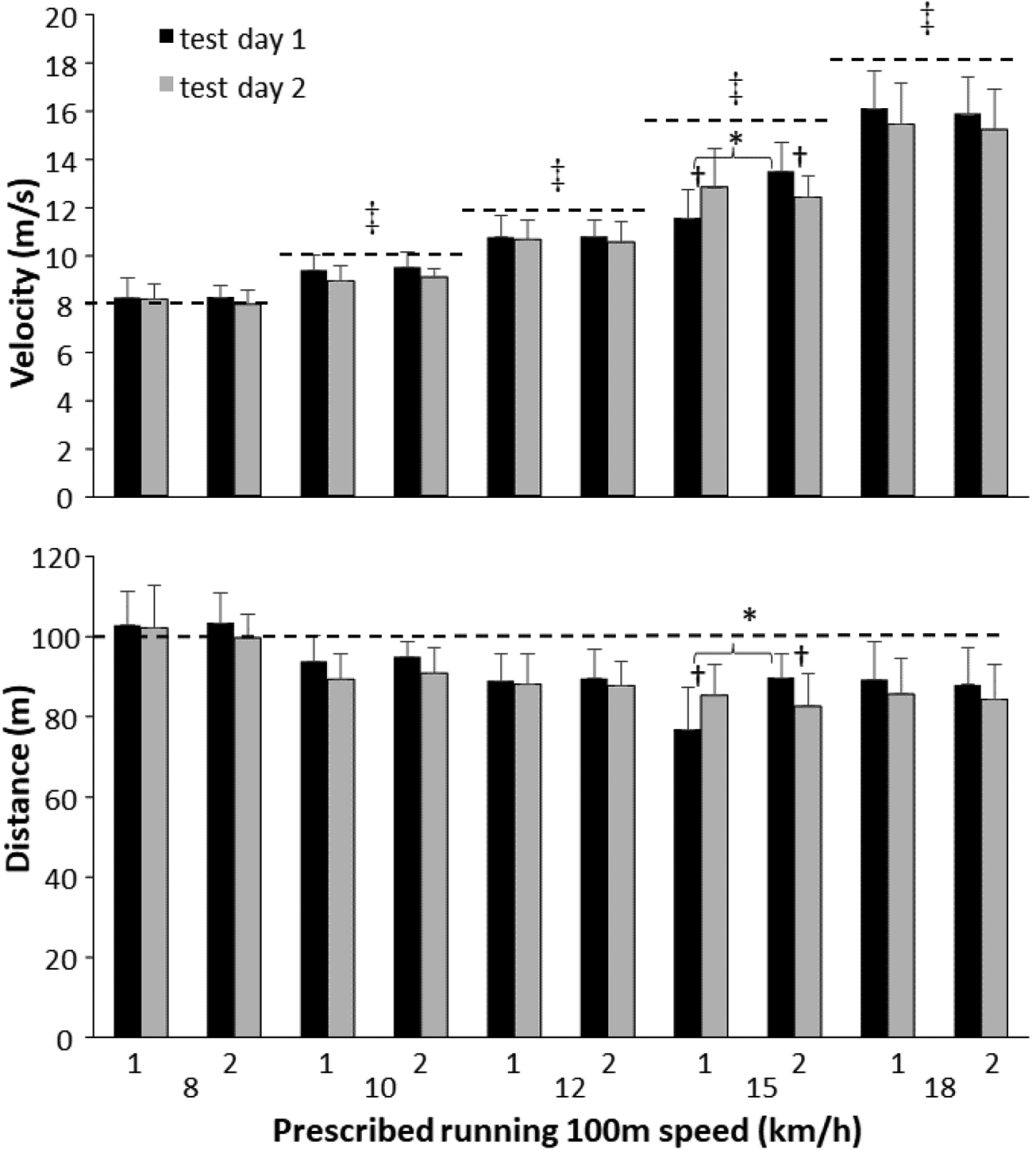
Means (±SD), velocities, and distances for each run at different intensities on each test day. * indicates a significant difference (*p* < 0.05) between the first and the second runs. † indicates a significant difference (*p* < 0.05) between the first and the second testing days. ‡ indicates a significant difference (*p* < 0.05) with actual velocity.

When analyzing the raw data on velocity and distance with the actual distance and corresponding speeds, a significantly lower velocity than the prescribed velocity for each speed was measured with Polar Team Pro (*t* ≥ 2.61, *p* ≤ 0.028), except at 8 km/h (*t* ≤ 1.79, *p* ≥ 0.11, Figure 2). On test day 1, the covered distance per prescribed speed (100 m) was significantly affected by speed (*F* = 30.0; *p* < 0.001; *η_p_*^2^ = 0.76), run (*F* = 20.9; *p* = 0.001; *η_p_*^2^ = 0.70), and interaction (*F* = 18.7; *p* < 0.001; *η_p_*^2^ = 0.67). On test day 2, only a significant effect of speed was found (*F* = 34.5; *p* < 0.001; *η_p_*^2^ = 0.81), but no significant effect of run and interaction (*F* ≤ 2.69; *p* ≥ 0.082; *η_p_*^2^ ≤ 0.25). On test day 1, the *post-hoc* comparison showed that the covered distance decreased with increasing speed ranging from 15 to 18 km/h. Furthermore, more distance was covered in the second 100 m run, which was only significant at 15 km/h (Figure 2). On test day 2, the covered distance decreased at increasing speeds from 8 to 10 km/h and 10 to 15 km/h (Figure 2).

**Figure 3:**
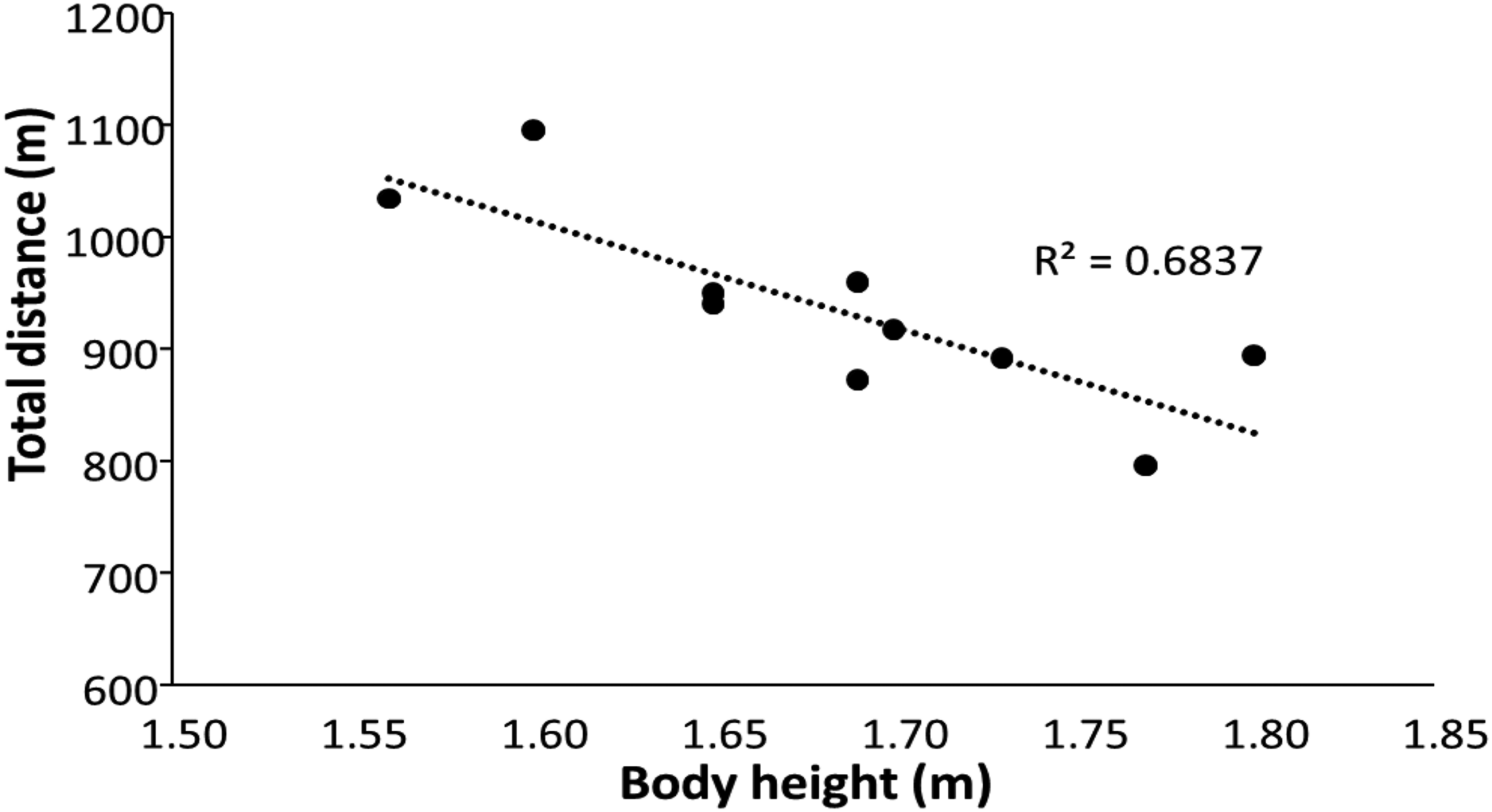
Relationship between body height and total distance covered during the test.

A repeated-measures design showed no significant differences in total running distances in the different speed categories and average velocity directly measured and calculated by Polar Team Pro between the two test days (*t* ≤ 1.47, *p* > 0.186, Table 2). However, when testing the raw data (distance and velocity per speed) between the two test days, a significant difference in distance and velocity between runs 1 and 2 was found only at 15 km/h, in which the running velocity was lower and measured at a shorter distance on test day 1 than that on test day 2. In run 2 at 15 km, the opposite was found (Figure 2). Day-to-day variation tested by the ICCs on the total distance and distances at different speeds between the two test days showed that the ICCs varied from 0.49 (poor) to 0.89 (good) reliability (Table 3).

**Table 3:** ICC between test days 1 and 2 for the total distance, distance at 0–11 km/h, distance at 11–19 km/h, and distance for each 100 m run.

	Distance			8 km		10 km		12 km		15 km		18 km	
Run	Total	0–11 km/h	11–19 km/h	1	2	1	2	1	2	1	2	1	2
ICC	0.70	0.66	0.75	0.75	0.72	0.59	0.49	0.78	0.74	0.78	0.89	0.50	0.60

ICC, intraclass correlation coefficient.

The random error viewed by the CV showed that CVs on the overall running distance, average velocity, and distances covered at different intensities, which were calculated by Polar Team Pro, varied from 6.7% to 31.6% (Table 2). The CVs calculated from the velocities at each speed, run, and test day were on an average of 8.5% over all runs and speeds and varied from 4.2% to 12.4% (Table 4).

**Table 4:** Coefficient of variation between the sensors on velocity and distance for each run, speed, and test day.

Speed	8 km		10 km		12 km		15 km		18 km	
Run	1	2	1	2	1	2	1	2	1	2
Test day 1										
Velocity	10.2	5.7	6.9	6.6	8.5	6.6	10.1	8.9	9.9	9.8
Distance	10.2	5.5	6.9	6.7	8.4	6.6	10.1	8.9	9.8	9.8
Test day 2										
Velocity	8.4	7.5	7.2	4.3	7.6	8.5	12.4	7.5	11.2	11.1
Distance	8.4	7.4	7.3	4.2	7.6	8.5	12.4	7.4	11.3	11.0

## Discussion

The aim of this study was to examine the reliability and validity of Polar Team Pro measurements of velocity and distance at different intensities in an indoor situation. The main finding revealed that Polar Team Pro underestimated the running distance and velocity, especially at higher speeds (15% and 6% at 15 and 18 km/h, respectively). Between test days, the measurements varied at different speeds, and coefficients of variations varied from 4.2% to 12.4%. However, a difference was found only at 15 km/h for the two runs between the two test days.

Polar Team Pro measured the total distance at 9.5%, which was shorter than it actually was. This was mainly caused by the shorter measured distances at speeds higher than 8 km/h. With increasing speed, the measured distance with the system became shorter until 15 km/h, in which only an average of 85 m was measured, which was 15% shorter than the actual distance. Thereby, the systematic error increased with increasing speed, but with no significant systematic error at 8 km/h (Figure 2). Since the distance was underestimated at higher speeds, the measured velocities at these speeds were lower, and thereby more distance was covered in the lower speed range (0–11 km/h) and less in the high range (11 to >19 km/h) than in reality (Table 2). This was probably caused by the inaccuracy of the accelerometers and algorithms in measuring and calculating the step length with increasing speed. With increasing speed, the step length increases, and an inaccuracy of approximately 0.3 m for each step can occur when sprinting at maximal velocity (11). Therefore, with increasing speed, longer steps are underestimated. This is also visible when comparing the body height with the total distance (Figure 3). Taller women generally have longer steps to cover the same distance, but with Polar Team Pro, it appears that the longer women run shorter, while they run the same distance. Thus, the validity of Polar Team Pro is high at 8 km/h and for women who are approximately 1.60 m tall. Our findings contradict those of Akyildiz et al. (4) and Huggins et al. (5), who investigated the accuracy and reliability of Polar Team Pro outdoors mainly in a straight line with acceptable reliability and validity. However, in outdoor settings, the system uses a GPS signal, which is not possible indoors, and the system then must rely on the IMU and the algorithms behind it to calculate the distances covered at different velocities during the rectangular run. Therefore, the IMU underestimates the distance in general during the indoor situation.

The variation in test–retest was apparent only at 15 km/h between runs 1 and 2. This finding could be explained by the following example. On test day 1 in the first trial, the participants did not know exactly at what pace 15 km/h was and ran too slowly (2–3 m). In the second run, they tried to compensate by running a bit too fast (reaching the finish cone 1–2 s too early). On test day 2, they became aware of the pace and thereby did not change the velocity from run 1 to run 2 (Figure 2). Our findings indicate that Polar Team Pro is accurate when measuring over different days. Although the ICC for the total distance was 0.70 (moderate reliability) and ICCs varied from 0.49 (poor) to 0.89 (good reliability) at different velocities (Table 2), the variation is probably caused by the small differences between the participants at each velocity. Even if they had to hold their distance between each other at every intensity, small changes in length between the participants (1–2 m) could have a large effect on the ICCs, as shown at 15 km between runs 1 and 2. To avoid this, each participant should have worn two Polar Team Pro belts to investigate the interunit accuracy better, as Akyildiz et al. (4) did. They showed similar ICCs for the total distance (0.63), but higher ICCs of 0.99 for the different speeds, but these were taken together.

The CVs for the total distance and average velocity were 6.7% on day 1% and 9.4% on day 2, which are acceptable, as the variation is below 10% (9). However, when specified per velocity, the CV varied from 4.2% to 12.4% (Table 3). For the speed categories 3–10.9 and 11–19 km/h, the CVs varied from 14% to 30% (Table 2). The high CVs for the speed categories can be explained by the fact that most units measured the velocities at 12 km/h and some units at 15 and under 11 km/h, and thereby the velocity was identified in another category, causing these high CVs. Furthermore, it was found that the CV between the units increased with increasing speed (Table 2), as also shown by increased systematic bias with these speeds. This indicates that the reliability decreases with higher speed in indoor situations, which was not the case in the studies by Akyildiz et al. (4) and Huggins et al. (5), who found the CV to be <5% in their outdoor testing situations.

The present study has some limitations. First, we only tested the system indoors and did not compare it directly in an outdoor setting, which could give information about how accurate the GPS is compared with the IMU algorithms. However, the manufacturer stated that one or two outdoor sessions are enough to calibrate the GPS with the IMU sensors to obtain accurate readings. Since the participants had worn the sensor for the prior 3 months during almost every indoor session, the system was expected to be accurately calibrated. Second, the participants were not of the same height and thus had different step lengths, which probably influenced the distance measurements. Our argumentation is supported by the fact that the most accurate data were with 1.6 m tall women. Furthermore, using two units for each participant instead of one increased the interunit variability and led to the generation of more accurate data. Finally, the participants ran after each other with the same distance between them. However, small changes in the distance between the participants could cause a large difference in the CV and ICC. In future studies, each participant should wear several sensors to avoid the possible difference (1–2 m) in the distance between participants and to measure the interunit accuracy more accurately.

## Conclusion

To the best of our knowledge, this is the first study to examine the reliability and validity of Polar Team Pro according to the measurements of distance and intensities during indoor conditions without the use of GPS. Based on the findings of the present study, we can conclude that Polar Team Pro underestimates the running distance and velocity when measuring a rectangular run at different speeds indoors. This underestimation takes place, especially at higher speeds, which is probably caused by the inaccuracy of the IMU algorithm that calculates the distance, as body height influences the distance and velocity measurements. The variability between the different units is, thereby, also influenced, causing variable CVs between the sensors. Test–retest variability was acceptable. Based on the findings of this study, practitioners should be cautious when measuring speed and distance using Polar Team Pro Sensors in indoor settings, as these measurements are underestimated with increasing speed.

## Data Availability

The raw data supporting the conclusions of this article will be made available by the authors, without undue reservation.
